# The influence of outcome expectancy on interpretation bias training in social anxiety: an experimental pilot study

**DOI:** 10.1186/s40814-023-01371-6

**Published:** 2023-08-17

**Authors:** Tonya Frommelt, Milena Traykova, Belinda Platt, Charlotte E. Wittekind

**Affiliations:** 1 Department of Child and Adolescent Psychiatry, Psychosomatics and Psychotherapy, LMU University Hospital Munich, Nußbaumstr. 5, Munich, 80336 Germany; 2 Department of Clinical Psychology and Psychotherapy, LMU Munich, Munich, Germany

**Keywords:** Social anxiety, Interpretation bias, CBM-I, Expectancy effects, Outcome expectations

## Abstract

**Background:**

Cognitive bias modification for interpretation (CBM-I) trainings have shown positive effects on interpretation bias in both active interpretation bias training conditions and structurally similar control conditions. Outcome expectations have been suggested to contribute to these placebo effects. The goal of this pilot experimental study was to test the feasibility of positive expectancy induction, to gain preliminary insight into whether this has implications for the efficacy of CBM-I training, and to assess the feasibility of recruitment and the overall study design.

**Methods:**

Socially anxious individuals aged 18 years and older received a single session (approx. 45 min) of either CBM-I or placebo training preceded by either a positive expectancy induction or no expectancy induction. We first tested whether the expectancy induction had modified participants’ expectations of training. We then explored the effects of CBM-I training and expectancy induction on interpretation bias. Finally, we assessed the feasibility of recruitment and further study procedures.

**Results:**

Due to pandemic-related difficulties, fewer participants were recruited than initially planned. Thirty-four (22 females and 12 males) participants were randomly assigned to one of four conditions (interpretation bias training + high expectancy = 10, interpretation bias training + no expectancy = 8, placebo training + high expectancy = 11, placebo training + no expectancy = 5). Participants in the positive expectancy condition had more positive expectations of the training (CBM-I or placebo) than participants in the no expectancy condition. We were unable to conduct the planned 2 × 2 × 2 analysis of interpretation bias due to the small sample size. When looking at these groups individually, we found that participants in the active training condition and participants in the high expectancy condition showed increases in positive interpretation bias and decreases in negative interpretation bias from pre- to post-training, while participants in the placebo and no expectancy conditions showed no change.

**Conclusions:**

These findings suggest that the expectancy manipulation utilized in this study may be adopted by future studies which investigate outcome expectations as an unspecific mechanism of CBM-I. Preliminary analyses suggest that participants’ expectations are likely to play a role in the effect of CBM-I training, although these effects require replication in a larger sample. Several observations about the study feasibility were made which could inform future trials.

**Trial registration:**

Retrospectively registered on the August 23, 2022, through the German Clinical Trials Register (DRKS00029768).

**Supplementary Information:**

The online version contains supplementary material available at 10.1186/s40814-023-01371-6.

## Key messages regarding feasibility

- Prior to this pilot study, it was uncertain whether the expectancy induction method developed for this study would induce the desired effect in a population of socially anxious individuals. Namely, whether those participants who were exposed to the framing text which depicted CBM-I as a treatment for social anxiety, would have higher expectations of the efficacy of CBM-I prior to the training. Furthermore, it was uncertain whether a successful expectancy manipulation would exert effects on the efficacy of CBM-I. Finally, it was uncertain whether recruiting socially anxious individuals to a clinic setting would be feasible for a larger target sample.
- The findings of this pilot study showed that the expectancy induction successfully altered participants’ outcome expectations of CBM-I training. Furthermore, initial insights were gained on the positive effect of outcome expectations on the efficacy of CBM-I. Finally, recruitment to the clinic setting was not considered ideal. It was not possible to distinguish whether this was due to the nature of the sample of socially anxious individuals or to the COVID-19 pandemic (or both).
- The expectancy induction utilized in the present pilot study may be adapted in future studies. Furthermore, the initial insights gained in terms of the positive effect of outcome expectations on the efficacy of CBM-I are encouraging and warrant further investigation of outcome expectations as an unspecific mechanism of CBM-I. Finally, it is suggested that future studies of this nature be conducted online due to recruitment barriers and further methodological issues.

## Background

### Cognitive biases in social anxiety

Symptoms of social anxiety often persist, despite subjects being confronted with social situations in the absence of negative feedback [1]. Cognitive models [2–4] suggest that this is because socially anxious individuals have socially specific information-processing biases, which maintain the disorder by making the individual more likely to perceive ambiguous social situations as threatening. The process of perceiving an ambiguous situation as threatening has been suggested to be influenced by both attention [5] and interpretation biases [6–10] for threat [11]. While it is possible to intervene at either the attentional or interpretational processing level [4], this study focuses on targeting interpretation biases, which has been shown to be a more effective method for reducing both cognitive biases and symptoms of social anxiety in socially anxious samples [12].

### Cognitive bias modification for interpretations (CBM-I) for social anxiety

Cognitive bias modification for interpretations (CBM-I) aims to alter participants’ interpretation bias (IB) by implicitly training them to interpret ambiguous social scenarios more positively and less negatively over a high number of brief, repetitive, and uninstructed trials [13] via their computer or smartphone. This contrasts with cognitive behavioral therapy (CBT), which targets negative IB through more explicit (effortful) cognitive restructuring. CBM-I may therefore be a useful supplementary tool to CBT for targeting IB in situations where less mental resources are available, such as when under stress or pressure [6]. Various meta-analyses with socially anxious individuals have shown that CBM-I cannot only reduce IB but can also reduce social anxiety symptoms [12, 14–16] and have a buffering effect on emotional reactivity to stress [9].

### Non-specific factors in CBM-I trainings

Various CBM-I studies have shown positive effects on IB not only in active CBM-I conditions but also in neutral CBM-I control conditions [17]. However, waitlist control conditions rarely show significant shifts in IB [14]. This phenomenon is not unique to CBM-I, a similar relationship between active therapy, placebo, and waiting list control conditions can be seen in the CBT literature [18]. The significant findings in neutral CBM-I control conditions raise the question of what may be driving the effects of CBM-I [16]. It is possible that unspecific training factors are causing a portion of the positive effects seen in the active and neutral control conditions, but not in the no-training control conditions. Both active and neutral control CBM-I conditions are executed in a similar manner [14] and structurally similar active and control conditions have been shown to elicit the greatest placebo effect [19]. In the CBM-I literature, various control conditions have been utilized. Some common examples of control conditions are those which provide participants with a 50/50 positive/negative resolution of ambiguity [20] or those with an emotionally neutral CBM-I task that does not encourage positive disambiguation of scenarios [10].

CBM-I requires minimal therapist contact as it is generally conducted via computer or smartphone [21], which means that unspecific factors involving the therapist (e.g., patient-therapist alliance and therapist empathy and support) are not likely to be major contributors to the placebo effects found in these trainings. Instead, outcome expectations have been discussed as a possible contributor to the placebo effects found in the CBM-I literature [16, 22]. Outcome expectations can be defined as the prognostic beliefs that subjects have about engaging in a specific type of treatment [23], can be evoked through factors such as a patient’s perception of how credible a treatment seems [24], and can be influenced by individual differences in age, sex, diagnosis [25], and symptom severity [26]. Studies vary greatly in terms of what participants are conveyed about CBM-I, with some studies making participants aware of the therapeutic benefits of CBM-I trainings via explicit instructions [16]. These instructions are not always documented in published manuscripts.

#### Outcome expectation as a non-specific factor in CBM-I

In a study examining the efficacy of a CBM-I training, results showed that participants in the control condition who believed they were in the active treatment condition had a positive treatment effect four times greater than control participants who were aware they were in the control condition [27]. Another study analyzed the relationship between highly anxious participants’ confidence in online CBM-I training (after receiving information about the rationale for CBM-I) and several outcome measures [28]. The researchers found a significant positive relationship between participants’ confidence in the training and drop-out rates as well as change in some IB measures, namely positive recognition ratings and scores on the Brief Body Sensations Interpretations Questionnaire (BBSIQ) [29]. However, no association was found between confidence in CBM-I and social anxiety symptoms. The authors noted that this insignificant result may be due to the high dropout rate, which significantly reduces the power of their study. In another study using both interpretation and attention bias modification trainings, a significant positive relationship was found between participants perceived ‘credibility and expectancy’ of training and the change in symptom severity at posttreatment and 2-week follow-up assessments [30]. Results such as these indicate the importance of outcome expectancy as a nonspecific factor that may contribute to the positive effects of CBM-I training. To the best of our knowledge, no previous studies have manipulated outcome expectancies between conditions, meaning, to date, a causal relationship between participant expectations and CBM-I trainings cannot be inferred.

### The current study

The original research question posed in this study was whether expectancy effects would have an influence on the efficacy of CBM-I in individuals with heightened social anxiety (retrospective registration: DRKS00029768). In line with previous findings, it was hypothesized that the CBM-I training in this study would have a positive effect on IB, social anxiety [14, 27, 31, 32], and emotional reactivity [9, 33] and that the greatest outcome success would be seen in those participants who received a positive expectancy induction [26, 34, 35]. In the current study, participants completed one session of active CBM-I or placebo training, which was either preceded by an expectancy induction or no expectancy induction. The main dependent variables were outcome expectations as measured using the Credibility and Expectancy Questionnaire (CEQ) [36] and two measures of IB, namely the Ambiguous Scenarios Recognition Task (AST-R [13]) and the Scrambled Sentence Task (SST [37]). Data on social anxiety symptoms and emotional reactivity were also collected; however, these data were not analyzed in the present pilot study.

Unfortunately, the COVID-19 pandemic made recruitment and testing extremely difficult so that the sample size of 168 participants could not be recruited in the available period. Nevertheless, the recruited sample of 34 participants was deemed sufficient to conduct exploratory research (1) testing the feasibility of the manipulation of participants’ pre-CBM-I-training expectations, (2) to gain initial insights into whether this influenced the efficacy of the CBM-I training, (3) to gain initial insights into the feasibility of modifying interpretation biases in socially anxious adults, (4) to assess the feasibility of recruiting socially anxious individuals to a clinic setting, and (5) to assess the feasibility of the overall study design. The revised goal of the present pilot study was, therefore, to form a basis for a larger randomized controlled trial (RCT).

The following criteria were used to determine the success of the pilot study and thus justify the commencement of a larger RCT: (1) a successful manipulation of participants` training expectations, such that participants in the high expectancy condition had greater outcome expectations of CBM-I training than those in the no expectancy condition, (2) initial insights that individuals in the high expectancy condition benefited more from the CBM-I training than those in the no expectancy condition, (3) initial insights that individuals in the CBM-I condition benefited more from the CBM-I training than those in the placebo condition, and the researchers should be of the opinion that (4) recruitment and (5) the overall study design are pragmatic and viable or easily adaptable.

The CONSORT Extension to Pilot and Feasibility Trials checklist was adhered to and can be viewed in the supplementary material (Additional file 1).

## Methods

### Participants

Eligible participants were adults aged 18 years and older with a good command of the German language and elevated social anxiety according to the Social Interaction Anxiety Scale (SIAS) [38]. A SIAS [38] score of ≥ 30 was chosen as the cutoff for inclusion/exclusion to this study, as this has been suggested to depict elevated social anxiety [38]. Participation was not possible for people currently suffering from a mental disorder other than social anxiety, as determined by the Mini International Neuropsychiatric Interview M.I.N.I [39], or those who were currently undergoing psychotherapeutic treatment. Participants were recruited via the university’s participant pool and online marketing (e.g., social networks). For compensation of study participation, subjects could choose between university test subject hours (ranging from 0.5 to 2.5, depending on how much time they spent in the study) and a merchandize voucher worth €20. During the first few months of recruitment, participants who chose the voucher option were placed in a raffle to win one of five vouchers. Later, to improve recruitment, each participant was offered a voucher for their participation. All participants provided written informed consent prior to study participation.

### Sample size

#### Original sample size calculation

The originally intended sample size was calculated based on a meta-analysis on therapy expectancy effects [34] which reported a small but significant positive effect of participants’ expectancies on treatment outcome,* d* = 0.24. Assuming a power of .80 and a significance level of .05 for two conditions and two measurement time points, for the comparison of social anxiety scores and measures of interpretation bias from pre- to post-training between the two outcome expectancy conditions a minimum sample size of *N* = 140 participants was calculated using the software G*Power [40]. Considering a dropout rate of approximately 20% can be expected in intervention studies, a total sample size of 168 participants (*n* = 42 per condition) was deemed suitable.

#### Modified sample size justification

When recruitment difficulties arose and it was no longer possible to achieve the necessary sample size for the originally planned analyses, we reconsidered how many participants would be necessary to test the feasibility outcomes of the present pilot study. Since sample size calculation is not required for feasibility studies, a pragmatic sample of 12 or more participants per group was considered appropriate as the sample of the present pilot study is representative of the target study [41, 42]. This means that the originally planned 2 × 2 × 2 analysis was not viable, as group sizes would be too small (i.e., interpretation bias training + high expectancy = 10, interpretation bias training + no expectancy = 8, placebo training + high expectancy = 11, placebo training + no expectancy = 5). In this pilot study, we therefore refrained from conducting the 2 × 2 × 2 analysis between training and expectancy groups across time and instead looked at the main effects of the training condition (interpretation bias training = 18, placebo training = 16) and expectancy induction (high expectancy = 21, no expectancy = 13) on interpretation bias separately within groups.

We conducted a separate power analysis when testing the viability of the expectancy manipulation. In a previous study [43], a similar expectancy manipulation elicited a large effect (*d* = 1.02) on participants’ outcome expectations. Assuming a power of .80 and a significance level of .05, a sample size of *N* = 10 would be necessary to detect the effect of the expectancy manipulation on participants’ outcome expectations using a dependant samples *t* test, according to the statistical software package G*Power.

### Study design and setting

**Fig. 1:**
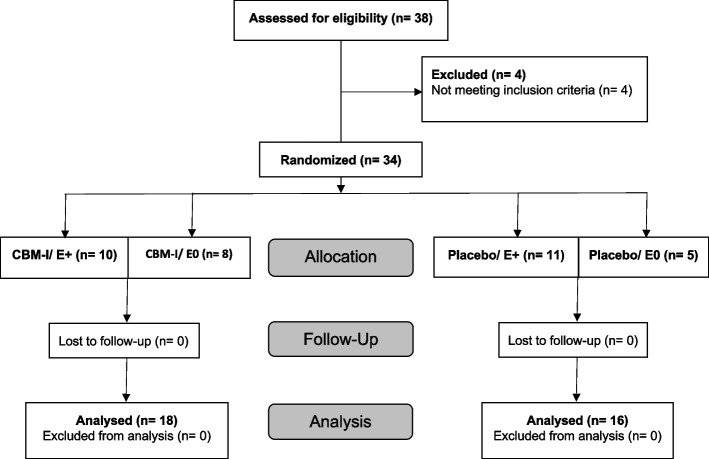
Participant flowchart *Note.* CBM-I cognitive bias modification for interpretation, E+ positive outcome expectation E0 no outcome expectation

The study design was a mixed design containing the two between-subject factors training (CBM-I vs. placebo) and outcome expectations (E+ vs. E0) and the within-subject factor time (pre- vs. post-training). The study had three assessment time points. These included pre-manipulation/training (measurements taken before training [t0]), post-manipulation/training (measurements taken directly after training [t1]), and a 1-week follow-up assessment (t2). See Fig. 1 for an overview of the different experimental groups and the number of participants in each group.

The study took place in two different locations at the LMU Munich. The initial 18 subjects were tested at the Department of Psychology, and the subsequent 20 participants were tested at the Department of Child and Adolescent Psychiatry, Psychosomatics and Psychotherapy of the University Hospital. The sessions were carried out by Clinical Psychology master’s students and supervised by post-doctoral researchers (CW, BP).

### Randomization and blinding

Sequence generation was conducted by CW, who was not involved in selecting and testing the participants. A computer-generated list of random numbers (nos. 1, 2, 3, and 4) corresponding to the four different experimental groups was created. To ensure allocation concealment, the experimenters were only made aware of a participant’s allocated group *after* study inclusion criteria were assessed and other baseline data were collected (i.e., immediately before the first study manipulation). For the initial 18 subjects sealed envelopes containing the group allocation codes were prepared and for the remaining 20 subjects the experimenter telephoned an individual in possession of the allocation sequence to obtain the relevant group code. Unfortunately, two participants were accidentally and unexplainably misallocated to the wrong intervention group. Whereas participants were kept blinded to their group allocation, the experimenters, and outcome assessors were not. Since recruitment ceased before the target sample size was obtained, the number of participants in the final groups analyzed in this study was not balanced (10:8:11:5).

### Expectancy manipulation

The study was advertised as a study on cognitive processes in social anxiety while refraining from using words such as “training” or “treatment” to avoid inducing an expectancy effect in those participants allocated to the no expectancy condition.

#### Positive expectancy induction (E+)

Immediately before the training began, participants in the E+ condition were presented with information on the computer screen, which aimed to induce a positive expectation of the training. The text informed participants that people with social anxiety tend to negatively interpret social situations and this in turn can trigger anxiety. The text then outlined how CBM-I trainings aim to counteract this negative IB by shifting it into a more adaptive direction, thus positively impacting symptoms of social anxiety. Participants were also told that CBM-I has been found to have positive effects on negative thoughts, confidence, positivity, and self-esteem [44]. The expectancy induction used in this study was based on a paradigm from a previous study, which found that framing an intervention study as a treatment study rather than a cognitive study to have superior effects on outcome measures [43]. A random selection of participants in both the active and placebo CBM-I conditions was allocated to the E+ condition. See supplementary materials (Additional file 2) for a full version of the English-translated expectancy induction text.

#### No expectancy induction (E0)

Participants in the E0 condition did not receive an expectancy induction before the training. They were informed that they would be completing a further cognitive task.

### Training conditions

**Fig. 2:**
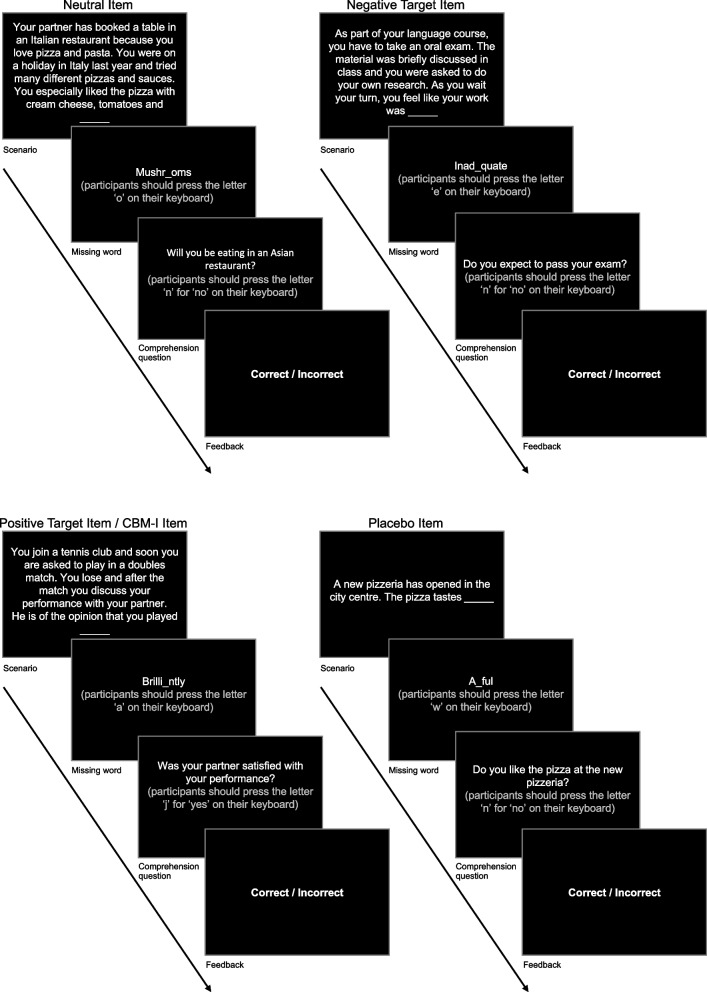
Item types *Note:* This figure depicts the different types of items present in the CBM-I and placebo training conditions. CBM-I cognitive bias modification for interpretation

Both the CBM-I and placebo training conditions involved administering a single session of the ambiguous scenarios task (AST) training paradigm [13]. Previous research has found that a single session of training can effectively alter socially anxious individuals cognitive biases [45]. The tasks were programmed and administered using Inquisit Lab version 5. In this task, participants were presented with various ambiguous scenarios where the last word in each scenario was missing (examples of such scenarios are presented in Fig. 2). Next, a fragmented version of the word which was missing from the scenario appeared. Participants had to complete the word fragment by pressing the missing letter on their keyboard. The missing word could resolve the ambiguous scenario in a negative or positive way (negative vs. positive valence scenarios). Participants were then presented with a control question that probed their knowledge of the scenario. Both conditions contained eight blocks (A to F) with 13 items/scenarios in each block.

Three items in each block were “neutral filler items.” These items had no social context, no emotional content, nor were they ambiguous. The purpose of these filler items was to make the CBM-I training less obvious [46]. Two items in each block were “target items” with social context. Within each block, one target item was resolved positively, and one was resolved negatively. These target items had two purposes. The first was to make the training induction less apparent by including some negative valence scenarios in the positive CBM-I condition. Secondly, these target items enabled the comparison of participants’ reaction times to positive versus negative valence social stimuli between training conditions across time [13]. However, reaction time data was not analyzed in the current pilot study, due to the small sample size. Both the CBM-I and placebo conditions contained the same neutral and the same target items. The remaining eight items in each block were specific to the CBM-I and placebo conditions.

#### CBM-I training

The eight unique items in each block of the CBM-I training condition described social situations which incorporated fears and symptoms typically experienced by people with heightened social anxiety. The scenarios were ambiguous, such that they could be resolved either negatively or positively. However, these items were always positively resolved in the CBM-I training condition, making them identical to positive valence target items.

#### Placebo training

The eight unique items in each block of the placebo training condition described everyday situations without social context. The scenarios were ambiguous, such that they could be resolved either negatively or positively. In the placebo condition, half of the scenarios were resolved positively and the other half were resolved negatively to avoid creating a bias for a positive or negative resolution of scenarios in general [46].

#### Stimulus materials

The item pool of this study consisted of a total of 164 items. The majority of items were obtained from previous work [13, 46, 47] and translated into German. The remaining items were created by the researchers involved in this study.

### Constructs and instruments

#### Measure of outcome expectation

To measure outcome expectation, a single question from the CEQ [36] “expectancy” subscale was presented immediately after the expectancy induction (E+ group only) and immediately before the training. This question was translated into German and modified to reflect the present study: “At this point, how helpful do you think participating in this study will be in reducing your social anxiety?” The item was rated on a 9-point Likert scale ranging from 1 (not at all) to 9 (very much). The purpose of using a single question was to avoid inducing an expectancy effect through the detailed questioning of study expectations. The test–retest reliability of the complete version of the CEQ [36] was found to be good using a 1-week time interval, with *r* = 0.82 for the expectancy factor [36].

#### Interpretation bias measures

By using both the AST-R [13] and the SST [37], generalization from the ambiguous scenario situation to a different type of task (the unscrambling of sentences) is enabled. Furthermore, different aspects of IB are captured [48], namely a more conscious and explicit aspect and a more automatic and implicit aspect, respectively [49].

The AST-R [13] consists of two parts. Part one of the AST-R [13] and the CBM-I training are identical, except that the valence of the ambiguous scenario is not resolved through the missing word in the AST-R [13].

**Fig. 3:**
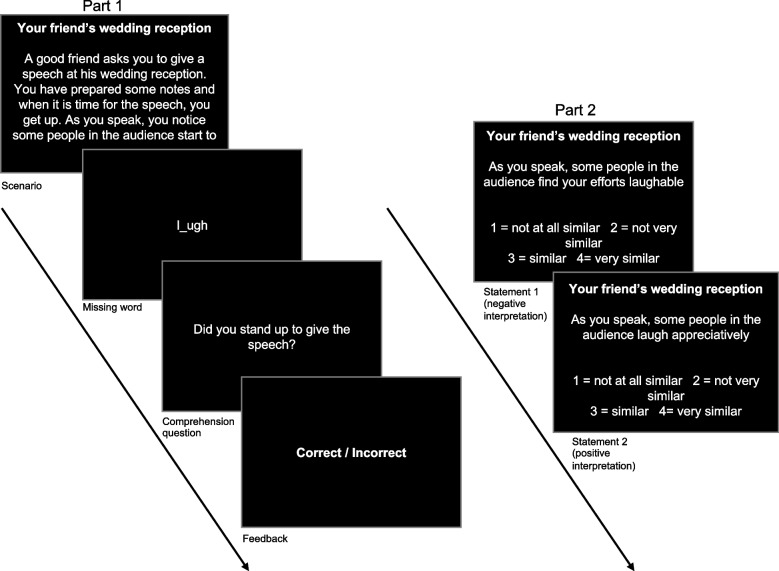
Design of the ambiguous scenario recognition task

In part two of the AST-R [13], participants are presented with one positive and one negative interpretation of the ambiguous scenario from part one. Participants are instructed to rate how similar in meaning the interpretations are to their associated scenario. Ratings are assessed on a 4-point Likert scale (1 = very different to 4 = very similar) [50]. In the AST-R [13], two separate summed scores are used to gage individuals’ positive and negative interpretation biases: (1) the sum of a participant’s scores on the positive interpretations and (2) the sum of a participant’s scores on the negative interpretations. See Fig. 3 for a depiction of the task.

**Fig. 4:**
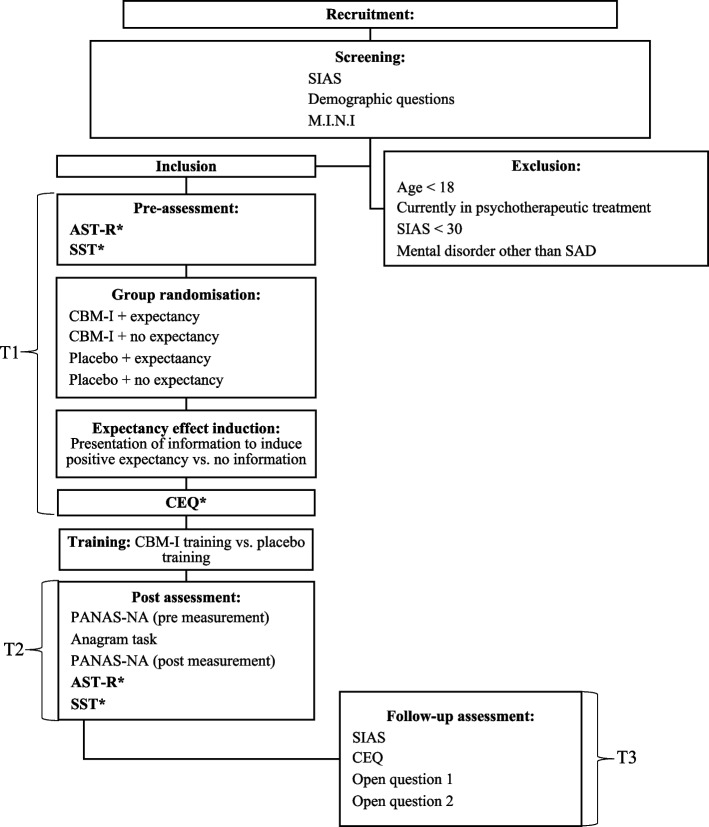
Study procedure flowchart *Note.* CBM-I cognitive bias modification for interpretation, SIAS Social Interaction Anxiety Scale [38], M.I.N.I. Mini International Neuropsychiatric Interview [39], SAD social anxiety disorder, SST scrambled sentence task [37], CEQ Credibility/Expectancy Questionnaire [36], AST-R Ambiguous Scenarios Recognition Task [13], PANAS-NA positive and negative affect schedule, negative affect subscale [51], Open question 1 “If you have just stated that your social anxiety has reduced since participating in the study, please describe the change in 1–2 sentences. If not, simply write ‘no’”; Open question 2 “What do you think was the purpose of the main cognitive task?”, *Instruments presented in bold were included in the analysis of this manuscript, while the remaining measures were not included due to the small sample size obtained

At both measurement time points (see Fig. 4), subjects were presented with seven social situations as well as seven negative and seven positive interpretations of each of the situations. Parallel versions of the AST-R [13] were used. Participants received both versions in randomized order. For the parallel test analyses of the AST-R [13] see the supplementary material (Additional file 3). The AST-R [13] was adopted from a previous study [50].

In the SST [37], participants are presented with six words and are instructed to build a sentence by using five of these words. Participants are informed that there would always be more than one possible option to build a correct sentence; however, they should only build one. Half of the word series contained emotional statements related to symptoms of social anxiety, where one positive and one negative statement could be formed. For example, “groups I confident am tense in” could be resolved as “in groups I am confident” or “in groups I am tense”. The other half of the word series contained neutral statements without emotional context. For example, “find pretty history I politics interesting” could be resolved as “I find politics pretty interesting” or “I find history pretty interesting”. Participants are informed that they would have 10 s to build a sentence and that they should try to work as quickly and accurately as possible. Only the emotional items are used to determine a person’s IB score, which was calculated as the proportion of negative responses out of all correct emotional responses.

In order to increase cognitive load and to minimize the resources available for strategy use [37, 52], participants simultaneously undergo a working memory load task where they should memorize a six-digit number. The number is presented at the beginning of the task for seven seconds and participants are asked to recite the number after they are finished unscrambling all the word-series.

At each measurement time point (see Fig. 4), subjects were presented with 20 randomized word series, ten with emotional valence and ten with neutral valence. Parallel versions of the SST [37] were used, and each participant received the two versions in randomized order. The items used in this study were modified from pre-existing German SST items [49], such that their content reflected the criteria of the DSM-5 (APA, 2013) for symptoms and fears typically occurring in people with social anxiety. For the parallel test analyses of the SST [37], see the supplementary material (Additional file 3).

#### Additional outcome measures

Emotional reactivity and social anxiety symptoms were also measured across time, using the positive and negative affect schedule (PANAS) [53] and the Social Interaction Anxiety Scale (SIAS) [38] These data were, however, not included in the analysis, due to the small sample size obtained. Information on these measures can be found in the supplementary materials section (Additional file 4).

### Procedure

A screening procedure was used to determine participants’ eligibility. Participants first filled out demographic questions and completed the SIAS [38]. The experimenter checked the SIAS [38] score to determine if the inclusion criterion was met (SIAS ≥ 30). It was also established, whether participants had prior knowledge and/or experience with CBM-I as this would exclude them from study participation. The experimenter then continued with the Mini-International Neuropsychiatric Interview [39] (M.I.N.I, [39]) to establish whether participants were suffering from any mental disorder other than social anxiety.

Eligible participants then completed the AST-R [13] and the SST [37] to assess their baseline IB scores. Subsequently, participants received their allocated expectancy induction (E+ vs. E0), followed by the adapted version of the CEQ [36] to measure their outcome expectations before training. Participants then continued with their assigned training task (CBM-I or placebo), which took approximately 45 min.

Immediately after the training (t1), participants filled out the PANAS-NA [51], undertook the anagram stressor task (see supplementary materials (Additional file 5)) for details on this task) and then filled out the PANAS-NA [51] for the second time, to determine whether groups differed in their emotional reactivity post-training. This was followed by the post-training assessment of IB using the AST-R [13] and SST [37]. The entire session took between 2 and 2.5 h.

The follow-up appointment (t2) took place 1 week later (±2 days). Participants again completed various questionnaires on the computer, beginning with the SIAS [38], followed by the adapted version of the CEQ [36] to retrospectively assess participants’ outcome expectations. Finally, participants were presented with two sequential open questions on the computer screen to determine their perceived change in social anxiety and their assumptions about the study goal. The follow-up appointment took approximately 10 min. Participants were fully debriefed and were given the option to receive a positive training if they had belonged to the placebo group. Finally, participants were thanked for their participation and given the option to choose between university subject hours or a voucher. See Fig. 4 for a flowchart of the study procedure.

### Statistical analyses

*Statistical Package for the Social Sciences* (IBM SPSS Statistics, Version 28) was used for all analyses. Two-tailed hypotheses with a significance level of *α* = .05 were tested and adjusted according to the Bonferroni procedure when multiple tests were performed on the same data. The normality of the data was assessed using the *p* value of the Shapiro–Wilk test, outliers were assessed using SPSS-generated boxplots, and heterogeneity of variance was assessed using the *p* value of Levene’s test (see supplementary material (Additional file 6) for the tests of assumptions).

An independent sample *t* test was used to determine whether the E+ and E0 groups differed in terms of their outcome expectations. For means of demonstration and because the data had already been collected, the main effects of the CBM-I training and the expectancy induction on IB were also reported by means of dependent sample *t* tests. However, data analyses were not performed on the remaining outcome measures (social anxiety [SIAS] and emotional reactivity [PANAS-NA]) due to inadequate power. Participants’ IB scores, measured via the AST-R [13] and the SST [37], were used as dependent variables. Separate Bonferroni corrections were applied to the AST-R [13], and the SST [37] data since these instruments may capture differing aspects of interpretation [49]. Four analyses were performed on the AST-R [13] data and two analyses were performed on the SST [37] data, meaning values exceeding the statistical threshold of *p* < .013 and *p* < .025, respectively, were interpreted as statistically significant.

## Results

Out of the 38 recruited participants, four participants were excluded from the study due to not meeting the criteria, three of which had SIAS [38] scores < 30 and one participant fulfilled the criteria for a depressive episode according to the M.I.N.I [39]. The recruited sample had a mean SIAS [38] score of = 52.21, *SD* = 10.17, and a range of = 44. The final sample consisted of 22 female (age: *M* = 26.27, *SD* = 5.35) and 12 male (age: *M* = 28.17, *SD* = 9.23) participants. See Fig. 1 for the flow of participants from eligibility assessment to statistical analysis.

The logic behind random assignment deems it unnecessary to assess differences in baseline scores between groups. However, because final group sizes in the present study were uneven due to recruitment difficulties, caution was taken and baseline differences between groups were assessed (see Table 1).

**Table 1:** Baseline scores between groups: means and standard deviations reported unless stated otherwise

	Condition					
	CBM-I/E+ (*n* = 10)	CBM-I/E0 (*n* = 8)	Placebo/E+ (*n* = 11)	Placebo/E0 (*n* = 5)	*P* Value	ES^a^
Sociodemo-graphic variables						
Sex (% of females)	60%	75%	55%	80%	.687	.21
Age (in years)	27.40 (8.21)	24.75 (3.96)	29.82 (7.61)	23.20 (4.15)	.241	.13
Pre-training measurements						
Social anxiety (SIAS t0)	49.50 (8.24)	53.88 (11.93)	51.09 (9.85)	57.40 (12.18)	.520	.07
Interpretation bias measures						
AST-R Neg. (t0)	20.20 (2.15)	19.38 (4.71)	19.45 (3.86)	19.40 (4.39)	.958	.01
AST-R Pos. (t0)	14.40 (3.34)	15.38 (4.07)	13.64 (2.20)	14.60 (2.51)	.692	.05
SST (t0)	65% (21%)	65% (34%)	63% (21%)	58% (23%)	.961	.01

*CBM-I* Cognitive bias modification of interpretation, *E*+ Positive outcome expectation, *E0* No outcome expectation, *SIAS t0* Social Interaction Anxiety Scale [38] scores at pre-training assessment, *AST-R Neg. (t0)* Ambiguous scenarios recognition task [13] negative interpretation scores at pre-training assessment, *AST-R Pos. (t0)* Ambiguous scenarios recognition task [13] positive interpretation scores at pre-training assessment, *SST (t0)* Scrambled sentence task [37] at pre-training assessment, *ES* Effect size

^a^For the variable sex, a *χ*^2^ test was used and Cramer’s V was used to measure the effect size. For all other variables, a one-way ANOVA was used, and partial eta-squared was used to measure the effect size

### Manipulation of outcome expectations

Findings revealed that there was a significant difference between E+ and E0, *t*(32) =  − 2.50, *p* = .018, 95% CI (− 3.36, − 0.35), with a large effect size of *d* =  − 0.85, 95% CI (− 1.57, − 0.12). Participants in the E0 condition had a mean outcome expectation score of 4.38 (*SD* = 2.47), and participants in the E+ condition had a mean outcome expectation score of 6.24 (*SD* = 1.84). These results are in line with a successful manipulation of outcome expectations between conditions.

### Influence of the expectancy induction on interpretation bias

Analyses showed that participants in the E+ condition had a significant increase in similarity ratings of positive interpretations, *t*(20) =  − 2.96, *p* = .008, 95% CI (− 5.12, − 0.88), *d* =  − 0.65, 95% CI (− 1.12, − 0.17), but no change in similarity ratings of negative interpretations from pre- to post-training, *t*(20) = 1.79, *p* = .089, 95% CI (− 0.33, 4.33), *d* = 0.39, 95% CI (− 0.06, 0.83), in the AST-R [13]. Participants in the E0 condition showed no change in their rating of positive, *t*(12) =  − 0.45, *p* = .661, *d* =  − 0.13, 95% CI (− 0.67, 0.42), or negative interpretations, *t*(12) = 0.68 *p* = .512, *d* = 0.19, 95% CI (− 0.36, 0.73), from pre- to post-training in the AST-R [13]. See Table 2 for the similarity ratings of positive and negative interpretations between expectancy conditions from pre- to post-training.

**Table 2:** AST-R: between training and expectancy conditions across measurement timepoints: means and standard deviations reported

*Condition*	**CBM-I* (n* = *18)***		**Placebo* (n* = *16)***	
*Time*	T0	T1	T0	T1
*Pos. interpretations*	14.83 (3.60)	18.22 (3.86)	13.94 (2.26)	14.50 (3.22)
*Neg. interpretations*	19.83 (3.43)	16.06 (4.52)	19.44 (3.88)	20.19 (4.09)
*Condition*	**E+ *(n* = *21)***		**E0 *(n* = *13)***	
*Time*	T0	T1	T0	T1
*Pos. interpretations*	14.05 (2.75)	17.05 (3.34)	15.00 (3.49)	15.54 (4.87)
*Neg. interpretations*	19.81 (3.12)	17.81 (4.65)	19.38 (4.41)	18.21 (5.06)

*AST-R* Ambiguous scenarios recognition task [13], *CBM-I* Cognitive bias modification for interpretation, *E*+ Positive outcome expectation, *E0* No outcome expectation, *Pos. interpretations* Similarity scores for positive interpretations, *Neg. interpretations* Similarity scores for negative interpretations, *T0* Pre-training assessment timepoint, *T1* Post-training assessment timepoint, *Diff*. Difference scores from t0 (pre-training assessment) to t1 (post-training assessment)

Moreover, analyses of the SST [37] data revealed that participants in the E+ condition did not have a significant decrease in the proportion of sentences which they resolved negatively from pre- to post-training, *t*(20) = 2.31 = , *p* = .032, 95% CI (0.01, 0.25), *d* = 0.50, 95% CI (0.04, 0.95), when applying the Bonferroni correction. Participants in the E0 condition showed no change in the proportion of sentences in which they resolved negatively in the SST [37] from pre- to post-training, *t*(12) = 1.29, *p* = .220, 95% CI (− 0.06, 0.22), *d* = 0.36, 95% CI (− 0.21, 0.92). See Table 3 for the proportions of negatively resolved sentences between expectancy conditions from pre- to post-training.

**Table 3:** SST: between training and expectancy conditions across measurement timepoints (means and standard deviations reported)

*Condition*	**CBM-I (*n* = 18)**		**Placebo (*n* = 16)**	
*Time*	T0	T1	T0	T1
*Proportion of negative solutions*	65.19% (26.99%)	46.30% (26.58%)	61.77% (21.15%)	59.44% (21.47%)
*Condition*	**E+ (*n*** **= 21)**		**E0** **(*n*** **= 13)**	
*Time*	T0	T1	T0	T1
*Proportion of negative solutions*	63.44% (21.50%)	50.57% (23.02%)	63.81% (28.79%)	55.58% (28.27%)

*SST* Scrambled sentences task [37], *CBM-I* Cognitive bias modification for interpretation, *E*+ Positive outcome expectation, *E0* No outcome expectation, *T0* Pre-training assessment timepoint, *T1* Post-training assessment timepoint, *Diff.* Difference scores from t0 (pre-training assessment) to t1 (post-training assessment)

### Influence of CBM-I training on interpretation bias

Analyses showed that participants in the CBM-I condition had a significant increase in similarity ratings of positive interpretations, *t*(17) =  − 3.16, *p* = .006, 95% CI (− 5.65, − 1.13), *d* =  − 0.69, 95% CI (− 1.16, − 0.21), and a significant decrease in similarity ratings of negative interpretations, *t*(17) = 3.39, *p* = .004, 95% CI (1.42, 6.13), *d* = 0.74, 95% CI (0.25, 1.22), from pre- to post-training in the AST-R [13]. Participants in the placebo condition showed no change in their rating of positive, *t*(15) =  − 0.52, *p* = .613, 95% CI (− 2.88, 1.76), *d* =  − 0.13, 95% CI (− 0.67, − 0.42), or negative interpretations, *t*(15) =  − 0.60, *p* = .556, 95% CI (− 3.41, 1.91), *d* =  − 0.15, 95% CI (− 0.69, − 0.40), from pre- to post-training in the AST-R [13]. See Table 2 for the similarity ratings of positive and negative interpretations between training conditions from pre- to post-training.

Moreover, analyses of the SST [37] data revealed that participants in the CBM-I condition had a significant decrease in the proportion of sentences which they resolved negatively from pre- to post-training, *t*(17) = 3.02, *p* = .008, 95% CI (0.06, 0.32), *d* = 0.71, 95% CI (0.22, 1.18), while participants in the placebo condition showed no change, *t*(15) = 0.47, *p* = .627, 95% CI (− 0.07, 0.12), *d* = 0.12, 95% CI (− 0.43, 0.66). See Table 3 for the proportions of negatively resolved sentences between training conditions from pre- to post-training.

## Discussion

The initial aim of this study was to investigate whether manipulating participants’ expectations of CBM-I training would influence the effectiveness of training on interpretation biases. Unfortunately, the COVID-19 pandemic made recruitment and testing extremely difficult such that the achieved sample size (*n* = 34) was lower than the goal sample size (*n* = 168). The achieved sample size was deemed sufficient to conduct exploratory analyses on the main effect of expectancy induction on pre-training expectations. Furthermore, the recruited sample of 34 participants was deemed sufficient to assess the feasibility of recruiting socially anxious individuals to a clinic setting and to assess the feasibility of the overall study design. The revised goal of the present pilot study was, therefore, to form a basis for a larger randomized controlled trial (RCT).

As anticipated, participants who received the positive expectancy induction had more positive expectations of the CBM-I training compared to participants who did not receive the expectancy induction. Due to the small sample size, we replaced the planned 2 × 2 × 2 mixed-design with exploratory within-participants *t* tests of pre- and post-changes and found that in general, participants in the high expectancy condition and in the CBM-I training condition showed greater improvements in IB than participants in the no expectancy or placebo conditions. Our experiences and findings make some important contributions to existing research in this field.

### Expectancy effect manipulation

The large effect size of *d* = 0.85 found for the expectancy manipulation developed in this study is in line with previous research which found superior effects in framing an intervention study as a treatment study rather than a cognitive study [43]. The successful manipulation of outcome expectations observed in this study indicates that the current methods may be used for future research which aims to fill the gap in knowledge on whether expectancy effects causally contribute to the efficacy of CBM-I.

### The effects of the expectancy manipulation and CBM-I training on interpretation bias

Furthermore, participants who were assigned to the E+ condition displayed a significant increase over time in positive interpretations in the AST-R [13] of a medium effect size. The change in negative interpretations over time was small in effect and non-significant. In line with expectations, participants in the E0 condition showed no shift in positive or negative interpretations over time in the AST-R [13]. For the SST [37], when applying the Bonferonni correction the change in negative interpretation bias over time was medium in effect and non-significant. As expected, participants in the E0 condition showed no change in their interpretation bias over time in the SST [37].

In line with expectations, participants in the CBM-I condition had a significant increase in positive interpretations and a significant decrease in negative interpretations over time of medium effect size, while participants in the placebo condition showed no change over in the AST-R [13]. Furthermore, as expected, participants in the CBM-I condition had a significant decrease in negative interpretation bias over time in the SST [37] of medium effect size, while participants in the placebo condition showed no change. Due to the small sample size, all *p* values should be viewed with caution, taking the increased risk of type 1 and type 2 errors into account. Furthermore, the small sample size could result in an over or under-estimate of the effect size.

The findings on the effect of the expectancy manipulation on interpretation bias are comparable with previous research which identified a significant positive relationship between participants’ confidence in a CBM-I training program and change in some, but not all, IB measures [28]. Furthermore, the findings are in line with previous observations that the treatment effect of an interpretation training program was much larger for participants in the control condition who believed that they were in the active treatment condition compared to those who were aware that they were in the control condition [27]. The findings of the effect of the CBM-I training condition on interpretation bias are in line with multiple studies on socially anxious individuals that show positive effects of CBM-I training on IB [14, 27, 31, 32]. Our findings on a small sample are promising in terms of a successful manipulation of participant expectancies of CBM-I and the effect which this has on IB as well as the successful shift in IB using CBM-I for socially anxious individuals. This pilot study contributes to current CBM-I research by providing initial insights into the role of expectancy effects in CBM-I trainings. The inconsistent findings in the CBM-I literature [17] leave open the question of which training-specific and non-specific mechanisms are contributing to the positive effects found in CBM-I. Research that focuses on determining which factors contribute to effects found in psychotherapeutic tools, such as CBM-I, can lead to improved theoretical models and can enhance the development of targeted interventions.

### Implications for future research

Several experiences gained during this pilot study could inform the feasibility of future larger RCT trials. For example, in addition to difficulties associated with the pandemic, we experienced difficulties recruiting participants with elevated social anxiety. This is because individuals who choose to participate must be willing to come to an unfamiliar location to talk to a stranger face-to-face about a sensitive topic. As we know from the literature, socially anxious individuals are less likely to attend therapy because of such factors. A related observation is that coming to an unfamiliar location could have influenced the experience participants have with the anagram stressor task, as it may seem less stressful after overcoming the initial hurdle of showing up to the study in the first place. While it is not possible to differentiate whether recruitment difficulties were predominantly due to the COVID-19 pandemic or the nature of the sample (or both), the authors suggest that future studies online may be beneficial as this could reduce barriers for socially anxious individuals to participate and represent less of an initial exposure experience.

A further valuable insight regarding recruitment was that when we adapted the study incentive by offering everyone the choice between study participation hours and a voucher (rather than study participation hours and being placed into a raffle to win one of 5 vouchers), we recruited more non-student participants. This suggests that by offering everyone the choice of a voucher we increased recruitment as the non-student participants may otherwise not have participated. Furthermore, adapting this method may boost the representativeness of future samples.

A further observation we made was that the time taken to respond to fragmented words with the correct missing letter in the main training task may have been contaminated by the time it took participants to search for the correct letter key. A solution for this problem may be to instruct participants to press the spacebar key when they know what the missing letter is, rather than searching for the key corresponding to the missing letter. This method has been implemented in previous research [50].

Furthermore, because the expectancy induction information text used in the present study mentioned the positive effects of CBM-I on both interpretation bias and on social anxiety, it would not have been possible to distinguish whether social anxiety improved as a result of changes in negative interpretation bias, which has been shown to have a mediating effect on social anxiety or due to the expected reduction in social anxiety mentioned in the expectancy induction information text. Future studies should therefore consider omitting the effects of CBM-I on social anxiety from the expectancy induction text to examine whether social anxiety was mediated by a reduction in negative interpretation bias.

### Limitations

The main limitation of this study was the small sample size, which prevented us from drawing valid conclusions about the interaction between participants’ expectations and the effects of CBM-I training. Despite not being able to address our initial aims, we nevertheless believe that the findings from the current study make a significant contribution to the existing literature (1) by demonstrating the large effect size associated with the expectancy manipulation in this clinical group, (2) by providing preliminary support from the first experimental study for the role of expectancy effects in CBM-I, and (3) by testing the feasibility of the study design in order to inform future larger-scale RCTs in this field. However, as the feasibility design and hypotheses were post hoc, even these findings are limited and may not be generalizable.

A further limitation of the current study was that the sample recruited may not be representative of the general population of socially anxious individuals. As mentioned above, the willingness of the participants in this study to take part in an extensive interaction with a stranger in a clinical setting during the COVID-19 pandemic may mean they are relatively psychologically resilient.

## Conclusion

The primary goal of this pilot study was to form a basis for a larger RCT by determining whether participants’ outcome expectations could be successfully manipulated using the expectancy induction method created for this study. Namely, by informing participants of the benefits of CBM-I on various health outcomes prior to the training. As expected, participants who received the positive expectancy induction gave higher ratings in their outcome expectations of the CBM-I training than participants who did not. This suggests that the expectancy manipulation utilized in this study may be adopted by future studies which aim to investigate outcome expectations as an unspecific mechanism of CBM-I. In general, participants in the CBM-I condition and participants who received the positive expectancy induction showed increases in positive IB and decreases in negative IB from pre- to post-training, while participants in the placebo and no-expectancy conditions showed no change. These findings on a small sample are promising and warrant further investigation of outcome expectations as an unspecific mechanism of CBM-I. In addition, they warrant the feasibility of the CBM-I training delivery methodology used in this study. Research which focuses on determining which factors contribute to effects found in CBM-I is vital for the further development and enhancement of this intervention and contribute to our understanding of the underlying theory.

Finally, this study assessed the feasibility of recruiting a sample of socially anxious individuals to a clinic as well as overall study procedures. Here, the authors point out that recruiting a larger target sample could present difficulties due to the nature of the sample. Additionally, coming to an unfamiliar location could have influenced the experience socially anxious participants have with the anagram stressor task, as it may seem less stressful after overcoming the initial hurdle of showing up to the study in the first place. Conducting similar studies online could therefore be beneficial. Moreover, offering each participant the choice between vouchers or study participation hours may not only improve recruitment but also the representativeness of the sample. The authors suggest two further methodological improvements, namely an adjustment of the response key used in the CBM-I training task and a slight modification of the expectancy induction text.

### Supplementary Information

**Additional file 1.** “CONSORT Extension to Pilot and Feasibility Trials checklist”.

**Additional file 2.** “Outcome Expectation Induction Text:” A full version of the English translated expectancy induction text.

**Additional file 3.** “Parallel Tests: Versions A and B of the AST-R [13] and the SST [37]”.

**Additional file 4.** “Descriptions of Outcome Measures not Included in the Analysis”: Emotional reactivity and social anxiety symptoms were also measured across time, using the Positive and Negative Affect Schedule (PANAS) [53] and the Social Interaction Anxiety Scale (SIAS) [38]. These two measures were not included in the main text as they were not included in the adapted pilot study reported in this paper.

**Additional file 5.** “Anagram Stressor Task”: To measure participants’ emotional reactivity after training, an anagram stressor task was included in the original study design.

**Additional file 6: Table 4.** “Assessing Violations of Assumptions Within Training Groups”. **Table 5.** “Assessing Violations of Assumptions Within Expectancy Group”.

## Data Availability

The datasets used and/or analyzed during the current study are available from the corresponding author on reasonable request.

## Abbreviations

CBM-I: Cognitive bias modification for interpretation
IB: Interpretation bias
CBT: Cognitive behavioral therapy
E+: Positive expectancy condition
E0: No expectancy condition
SIAS: Social Interaction Anxiety Scale
M.I.N.I.: Mini International Neuropsychiatric Interview
AST-R: Ambiguous scenarios recognition task
SST: Scrambled sentence task
CEQ: Credibility/Expectancy Questionnaire
PANAS-NA: Positive and negative affect schedule, negative affect subscale
T0: Pre-assessment
T1: Post-assessment
T2: One-week follow-up assessment
