# Supplementary material for: The influence of outcome expectancy on interpretation bias training in social anxiety: an experimental pilot study
Source: Pilot Feasibility Stud. 2023 Aug 17;9:144. doi: 10.1186/s40814-023-01371-6 (PMC10433573; doi:10.1186/s40814-023-01371-6)
Supplement: Supplementary file 2 — Additional file 2. “Outcome Expectation Induction Text:” A full version of the English translated expectancy induction text. [file 40814_2023_1371_MOESM2_ESM.pdf]

Additional File 2

Outcome Expectation Induction Text

Dear participant,

In a moment you will receive a training program that aims to reduce symptoms of social anxiety.

People with increased social anxiety often interpret social situations in a negative way. This can include negative thoughts and evaluations such as, "I must have done/said something embarrassing," or "I hope no one talks to me." Such evaluations and thoughts also affect behaviour and can trigger and reinforce discomfort, insecurity, or even anxiety.

There are several methods to counteract anxiety and insecurity in social situations. Recent developments in this field include computer programs which aim to change negative thoughts and evaluations.

Cognitive Bias Modification of Interpretation (CBM-I) is a computer-based training programs that addresses negative thoughts and interpretations in social situations. The goal of a CBM-I training is to shift individuals' interpretations of social situations in a more functional direction through a high number of short tasks on the computer, thereby having a positive impact on social anxiety. CBM-I trainings have been widely used in the past to change dysfunctional appraisals and have been shown to have positive effects on various outcome measures.

For example, numerous studies have shown that with a single session of CBM-I training, participants' attitudes in social situations were more positive. Furthermore, participants reported fewer negative thoughts, were more positive and their self-confidence increased. In addition, CBM-I training reduced participant insecurity and discomfort in various social situations, including at work, in groups and in relationships.

The following is a session of such CBM-I training for individuals with social anxiety.
