# Supplementary material for: The influence of outcome expectancy on interpretation bias training in social anxiety: an experimental pilot study
Source: Pilot Feasibility Stud. 2023 Aug 17;9:144. doi: 10.1186/s40814-023-01371-6 (PMC10433573; doi:10.1186/s40814-023-01371-6)
Supplement: Supplementary file 3 — Additional file 3. “Parallel Tests: Versions A and B of the AST-R [13] and the SST [37]”. [file 40814_2023_1371_MOESM3_ESM.pdf]

Additional File 3

Parallel Tests: Versions A and B of the AST-R (1) **and the SST** (2)

Parallel tests were performed to determine whether the versions A and B of each interpretation bias instrument reported in this manuscript differed at pre-assessment (t0). Independent *t*-tests revealed that the two versions of the AST-R (1) did not significantly differ (negative interpretations: version A: $M = 19.94$, $SD = 3.82$, version B: $M = 19.35$, $SD = 3.46$, $t(32) = 0.47$, $p = .641$; positive interpretations: version A: $M = 13.94$, $SD = 3.63$, version B: $M = 14.88$, $SD = 2.32$), $t(32) = -0.90$, $p = .374$). Furthermore, independent *t*-tests revealed that versions A and B of the SST (2) used in this study, did not differed from one another (version A: $M = 65\%$, $SD = 26\%$; version B: $M = 62\%$, $SD = 23\%$), $t(32) = 0.42$, $p = .678$).

1. Mathews A, Mackintosh B. Induced emotional interpretation bias and anxiety. J Abnorm Psychol. 2000;109(4):602–15.
2. Wenzlaff RM, Bates DE. Unmasking a cognitive vulnerability to depression: how lapses in mental control reveal depressive thinking. J Pers Soc Psychol. 1998 Dec;75(6):1559–71.
