# Supplementary material for: The influence of outcome expectancy on interpretation bias training in social anxiety: an experimental pilot study
Source: Pilot Feasibility Stud. 2023 Aug 17;9:144. doi: 10.1186/s40814-023-01371-6 (PMC10433573; doi:10.1186/s40814-023-01371-6)
Supplement: Supplementary file 4 — Additional file 4. “Descriptions of Outcome Measures not Included in the Analysis”: Emotional reactivity and social anxiety symptoms were also measured across time, using the Positive and Negative Affect Schedule (PANAS) [53] and the Social Interaction Anxiety Scale (SIAS) [38]. These two measures were not included in the main text as they were not included in the adapted pilot study reported in this paper. [file 40814_2023_1371_MOESM4_ESM.pdf]

## Additional File 4

**Descriptions of Outcome Measures not Included in the Analysis**

The Positive and Negative Affect Schedule (PANAS) is a brief self-report questionnaire used to measure positive and negative affect. The PANAS (1) consists of 20 items, which include 10 positive affect items (PA subscale) and 10 negative affect items (NA subscale). In this study, the NA subscale of the German version of the PANAS (2) was used to measure participants' emotional reactivity in response to the anagram task. The PANAS-NA (2) was administered both immediately before and after the anagram task (at t1). The NA subscale includes descriptors items such as, "distressed", "upset", "scared" and "afraid". Participants were asked how much each descriptor applied to them in the present moment. Responses were measured on a 5-point Likert-scale ranging from 1 (very slightly or not at all) to 5 (extremely). The internal consistency of the German version of the PANAS NA (2) is good, α = 0.86 (NA subscale), α = 0.85 (PA subscale). The internal consistency of the PANAS NA in this study was found to be *a* = 0.855 at measurement 1 and *a* = 0.866 at measurement 2 and can therefore also be interpreted as good (3). The German version of the PANAS NA (2) has previously been tested for external validity, where a significant correlation was found between the NA subscale and the personality trait 'anxiousness' ($r$ = 0.58).

The SIAS (4) measures cognitive and affective aspects of anxiety in social interactions and was used to determine the effect of the CBM-I training on social anxiety. Participants should indicate to what extent certain statements applied to them in the past seven days. The SIAS (4) consists of 20 items and each item is measured on a 5-point Likert scale ranging from "not at all characteristic or true of me" to "extremely characteristic or true of me." The German version of the SIAS (5), which was used in this study, has previously been found to have very good internal consistency, α = 0.94 (5), 0.93 (6). The internal

consistency of the SIAS in the present study was *a* = .793 at pre assessment, and *a* = .869 at post assessment. These results can be interpreted as acceptable and good, respectively (3). Furthermore, the German version of the SIAS (5) has previously been found to be significantly correlated with the Symptom Checklist 90 Revision (SCL- 90-R) subscale 'Insecurity in Social Contact,' thus showing sufficient convergent validity ($r = 0.71$, $p < 0.01$). The SIAS (5) has been reported to have good psychometric properties overall (5,6), it has a short completion time of approximately 10 minutes (5) and has been found to be sensitive in measuring therapeutic effects from pre to post assessment, t = 6,5, p < 0,01, d = 0,77 (6).

## References


1. Watson D, Clark LA, Tellegen A. Development and validation of brief measures of positive and negative affect: the PANAS scales. J Pers Soc Psychol. 1988;54(6):1063.
2. Krohne HW, Egloff B, Kohlmann CW, Tausch A. Untersuchungen mit einer Deutschen version der "Positive and Negative Affect Schedule" (PANAS). Diagnostica. 1996;42(2):139–56.
3. Nunnally JC. Psychometric theory. 2nd ed. New York: McGraw-Hill; 1978.
4. Mattick RP, Clarke JC. Development and validation of measures of social phobia scrutiny fear and social interaction anxiety. Behav Res Ther. 1998;36(4):455–70.
5. Stangier U, Heidenreich T, Berardi A, Golbs U, Hoyer J. Die Erfassung sozialer Phobie durch Social Interaction Anxiety Scale (SIAS) und die Social Phobia Scale (SPS). Z Klin Psychol Psychother. 1999;28(1):28–36.
6. Heinrichs N, Hahlweg K, Fiegenbaum W, Frank M, Schröder B, Von Witzleben I. Validität und Reliabilität der Social Interaction Anxiety Scale (SIAS) und der Social Phobia Scale (SPS). Verhaltenstherapie. 2002;12:26–35.
