# Supplementary material for: The influence of outcome expectancy on interpretation bias training in social anxiety: an experimental pilot study
Source: Pilot Feasibility Stud. 2023 Aug 17;9:144. doi: 10.1186/s40814-023-01371-6 (PMC10433573; doi:10.1186/s40814-023-01371-6)
Supplement: Supplementary file 5 — Additional file 5. “Anagram Stressor Task”: To measure participants’ emotional reactivity after training, an anagram stressor task was included in the original study design. [file 40814_2023_1371_MOESM5_ESM.pdf]

Additional File 5

Anagram Stressor Task

An anagram stressor task was used to investigate the impact of the CBM-I training on participants' emotional reactivity. Previous CBM studies have shown that anagram tasks are an effective means of stress-induction (1). The task comprised 15 anagrams made up of six letters each. Three of the anagram tasks were easy, six were difficult and six were unsolvable. Participants had 10 seconds to solve each anagram. Moreover, before beginning the anagram task, the experimenter informed participants that they would be filmed during the task. The purpose of this was to further increase participants' stress levels. At the end of the task, all participants were informed that their performance had been below average, with the aim of again intensifying participants' stress levels. The items used in this study were adopted from a pre-existing German anagram task (2).

References

1. Salemink E, van den Hout M, Kindt M. Effects of positive interpretive bias modification in highly anxious individuals. J Anxiety Disord. 2009;23(5):676–83.
2. Koppe K, Rothermund K. Let it go: Depression facilitates disengagement from unattainable goals. J Behav Ther Exp Psychiatry [Internet]. 2017;54:278–84. Available from: http://dx.doi.org/10.1016/j.jbtep.2016.10.003
