# Supplementary material for: The influence of outcome expectancy on interpretation bias training in social anxiety: an experimental pilot study
Source: Pilot Feasibility Stud. 2023 Aug 17;9:144. doi: 10.1186/s40814-023-01371-6 (PMC10433573; doi:10.1186/s40814-023-01371-6)
Supplement: Supplementary file 6 — Additional file 6: Table 4. “Assessing Violations of Assumptions Within Training Groups”. Table 5. “Assessing Violations of Assumptions Within Expectancy Group”. [file 40814_2023_1371_MOESM6_ESM.pdf]

Additional File 6

Table 4

Assessing Violations of Assumptions Within Training Groups

Variable	*CBM-I* Outliers	Normal Distribution (*p*-Value Shapiro-Wilk-Test)	*Placebo* Outliers	Normal Distribution (*p*-Value Shapiro-Wilk-Test)	Homogeneity of Variance (*p*-Value of Levene's Test)
AST-R Pos. T0	No	.354	No	.328	.088
AST-R Neg. T0	No	.043*	No	.077	.512
AST-R Pos. T1	Yes (not extreme)	.967	Yes (not extreme)	.229	.521
AST-R Neg. T1	No	.651	Yes (not extreme)	.072	.500
SST T0	No	.099	Yes (not extreme)	.599	.152
SST T1	Yes (not extreme)	.497	No	.111	.699

Note: The following table depicts the results of tests conducted to assess violations of assumptions for the data used to assess the influence of the CBM-I training; * = a violation of an assumption (in the case of outliers it would indicate an extreme outlier); Outliers were assessed using SPSS generated boxplots; The *p* value of the Shapiro-Wilk test was used to assess whether the data were normally distrusted; The *p* value of Levene's test was used to assess the homogeneity of variance; AST-R Neg. t0 / t1= Ambiguous Scenarios Recognition Task (1) negative interpretations at pre/ post training assessment timepoint; AST-R Pos. t0/t1 = Ambiguous Scenarios Recognition Task (1) positive interpretations at pre/post training assessment timepoint; SST t0/t1 = Scrambled Sentence Task (2) at pre/post training assessment timepoint.

Table 5

Assessing Violations of Assumptions Within Expectancy Groups

Variable	*High Expectancy (E+)* Outliers	Normal Distribution (*p*-Value Shapiro-Wilk-Test)	*No Expectancy (E0)* Outliers	Normal Distribution (*p*-Value Shapiro-Wilk-Test)	Homogeneity of Variance (*p*-Value of Levene's Test)
CEQ	No	.280	No	.234	.106
AST-R Pos. T0	No	.632	Yes (not extreme)	.053	.484
AST-R Neg. T0	Yes (not extreme)	.418	No	.645	.041*
AST-R Pos. T1	No	.046*	No	.627	.180
AST-R Neg. T1	No	.620	No	.093	.865

SST T0	No	.519	No	.507	.185
SST T1	Yes (not extreme)	.537	No	.940	.409

Note: The following table depicts the results of tests conducted to assess violations of assumptions for the data used to assess the influence of the expectancy induction; * = a violation of an assumption (in the case of outliers it would indicate an extreme outlier); Outliers were assessed using SPSS generated boxplots; The *p* value of the Shapiro-Wilk test was used to assess whether the data were normally distrusted; The *p* value of Levene's test was used to assess the homogeneity of variance; AST-R Neg. t0 / t1= Ambiguous Scenarios Recognition Task (1) negative interpretations at pre/ post training assessment timepoint; AST-R Pos. t0/t1 = Ambiguous Scenarios Recognition Task (1) positive interpretations at pre/post training assessment timepoint; SST t0/t1 = Scrambled Sentence Task (2) at pre/post training assessment timepoint.

References

1. Mathews A, Mackintosh B. Induced emotional interpretation bias and anxiety. J Abnorm Psychol. 2000;109(4):602–15.
2. Wenzlaff RM, Bates DE. Unmasking a cognitive vulnerability to depression: how lapses in mental control reveal depressive thinking. J Pers Soc Psychol. 1998 Dec;75(6):1559–71.
